# Effect of prednisone on type I interferon signature in rheumatoid arthritis: consequences for response prediction to rituximab

**DOI:** 10.1186/s13075-015-0564-y

**Published:** 2015-03-23

**Authors:** Tamarah D de Jong, Saskia Vosslamber, Marjolein Blits, Gertjan Wolbink, Mike T Nurmohamed, Conny J van der Laken, Gerrit Jansen, Alexandre E Voskuyl, Cornelis L Verweij

**Affiliations:** Department of Pathology, VU University Medical Center, P.O. Box 7075, 1007 MB Amsterdam, The Netherlands; Department of Rheumatology, VU University Medical Center, Amsterdam, The Netherlands; Jan van Breemen Institute | Reade, Amsterdam, The Netherlands

## Abstract

**Introduction:**

Elevated type I interferon (IFN) response gene (IRG) expression has proven clinical relevance in predicting rituximab non-response in rheumatoid arthritis (RA). Interference between glucocorticoids (GCs) and type I IFN signaling has been demonstrated *in vitro*. Since GC use and dose are highly variable among patients before rituximab treatment, the aim of this study was to determine the effect of GC use on IRG expression in relation to rituximab response prediction in RA.

**Methods:**

In two independent cohorts of 32 and 182 biologic-free RA patients and a third cohort of 40 rituximab-starting RA patients, peripheral blood expression of selected IRGs was determined by microarray or quantitative real-time polymerase chain reaction (qPCR), and an IFN-score was calculated. The baseline IFN-score was tested for its predictive value towards rituximab response in relation to GC use using receiver operating characteristics (ROC) analysis in the rituximab cohort. Patients with a decrease in disease activity score (∆DAS28) >1.2 after 6 months of rituximab were considered responders.

**Results:**

We consistently observed suppression of IFN-score in prednisone users (PREDN^+^) compared to non-users (PREDN^−^). In the rituximab cohort, analysis on PREDN^−^ patients (n = 13) alone revealed improved prediction of rituximab non-response based on baseline IFN-score, with an area under the curve (AUC) of 0.975 compared to 0.848 in all patients (n = 40). Using a group-specific IFN-score cut-off for all patients and PREDN^−^ patients alone, sensitivity increased from 41% to 88%, respectively, combined with 100% specificity.

**Conclusions:**

Because of prednisone-related suppression of IFN-score, higher accuracy of rituximab response prediction was achieved in PREDN^−^ patients. These results suggest that the IFN-score-based rituximab response prediction model could be improved upon implementation of prednisone use.

## Introduction

Rheumatoid arthritis (RA) is a systemic autoimmune disease characterized by chronic joint inflammation which may lead to cartilage and bone destruction. It is a heterogeneous disease, as reflected by differences in severity, pathogenesis and treatment outcome. From diagnosis onwards, RA patients often receive immunosuppressive treatment with non-biologic disease-modifying anti-rheumatic drugs (DMARDs) and/or glucocorticoids (GCs). When patients no longer benefit from the non-biologic therapy, they usually start on treatment with biologics, such as TNFα-blockers and B-cell depletion therapy using rituximab (RTX) [1]. Approximately 30% to 50% of patients do not achieve a favorable response to biologics. To increase treatment efficacy and to develop personalized treatment, predictors of therapy response are needed.

Independent studies have shown that activation of the type I interferon (IFN) system is associated with the clinical outcome of RTX therapy [2,3]. This so-called ‘IFN signature’ represents a response program consisting of genes that are activated by type I IFNs and is present in approximately 50% of RA patients [4]. Induction of type I IFN response genes (IRG) is triggered via activation of the JAK-STAT signaling pathway, more specifically via JAK1, TYK2, STAT1 and STAT2, followed by recruitment of IRF9 and formation of the ISGF3 transcription factor complex [5]. It was shown that patients with a good response to RTX have low IRG expression prior to the start of treatment, whereas non-responders display relatively high IRG expression. Potential clinical utility of IRG expression reflected as an IFN-score to predict the clinical outcome of RTX treatment was demonstrated by an area under the receiver operating characteristics (ROC) curve of 87% [3]. Hence, knowledge of IRG expression in a RA patient before the start of RTX treatment is of crucial importance to predict the success of the clinical outcome.

It has been reported that GCs can interfere with the type I IFN system by modulation of IFN induction as well as downstream IFN signaling [6,7]. GCs were initially prescribed to RA patients in high doses (≥10 mg/day) to suppress flares of inflammation, but nowadays long-term treatment with low-dose GCs is commonly used as well [8]. Since use and dose of GCs are highly variable among patients prior to the start of treatment with RTX [2,3], we aimed to determine what the effect of GC use is on IRG expression in relation to the clinical response to RTX.

## Methods

### Patients

This study consisted of three independently collected cohorts. All patients fulfilled the revised American College of Rheumatology (ACR) 1987 criteria for RA diagnosis [9]. Patient characteristics are shown in Table 1. Cohort I included 32 RA patients of whom 6 patients were treated with the GC prednisone, as previously reported [4]. Cohort II was recruited from Jan van Breemen Research Institute | Reade, Amsterdam, the Netherlands, and consisted of 182 RA patients, of whom 52 patients received prednisone. The patients in these two cohorts had not been on any biologic treatment. Cohort III was recruited from Jan van Breemen Research Institute | Reade and the VU University Medical Center, Amsterdam, the Netherlands, and consisted of 40 RA patients, of whom 27 patients were using prednisone [3]. These 40 patients were candidates for RTX therapy because of their high disease activity (disease activity score in 28 joints (DAS28) >3.2) despite DMARD treatment and previous anti-TNF therapy. At the moment of blood collection, patients were off anti-TNF therapy for at least four weeks and had not received their first RTX dose yet. The clinical response to RTX was determined based on the change in DAS28 after six months of therapy; patients with a ∆DAS28 > 1.2 were considered responders [10]. All patients provided written informed consent and the participating clinics received approval by the medical ethics committees of the Academic Medical Center, the VU University Medical Center and the Jan van Breemen Research Institute | Reade.

**Table 1:** **Patient characteristics of the included cohorts**

**Characteristics**	**Cohort I**	**Cohort II**	**Cohort III (rituximab)**
	**Number = 32**	**Number = 182**	**Number = 40**
**Demographics**			
Age, years	49 ± 10	54 ± 12	57 ± 10
Female, number (%)	22 (69)	135 (75)	34 (85)
**Disease characteristics**			
Disease duration, years	7.5 ± 8.9	9.5 ± 10.2	11.0 ± 9.5
Disease activity (DAS28)	5.4 ± 1.3	5.1 ± 1.2	5.8 ± 1.1
ESR, mm/hour	29.3 ± 22.2	24.5 ± 18.0	29.2 ± 23.8
CRP, mg/L	18.8 ± 19.4^a^	17.8 ± 22.1	17.7 ± 17.7
Erosions, number (%)	24 (75)	131 (72)	28 (72)
IgM RF positive, number (%)	28 (88)	130 (71)	27 (68)
ACPA positive, number (%)	26 (87)^b^	129 (75)^c^	29 (73)
**Medication**			
Current prednisone use, number (%)	6 (19)	52 (29)	27 (68)
Prednisone dosage, mg/day	8 ± 2	7.2 ± 3.5	6.75 ± 6.0
Current MTX use, number (%)	25 (78)	152 (84)	26 (65)
MTX dosage, mg/week	21.2 ± 7.1	21.0 ± 6.3	18.7 ± 8.2
Current SSZ use, number (%)	N/A	27 (16)^d^	7 (18)
Current HCQ use, number (%)	N/A	35 (20)^d^	5 (13)

^a^Data missing for 7 of the 32 patients; ^b^data missing for 2 of the 32 patients; ^c^data missing for 9 of the 182 patients; ^d^data missing for 9 of the 182 patients. Continuous variables are presented as mean with standard deviation. ACPA, anti-cyclic citrullinated protein antibody; CRP, C-reactive protein; ESR, erythrocyte sedimentation rate; HCQ, hydroxychloroquine; MTX, methotrexate; N/A, not applicable;RF, rheumatoid factor; SSZ, sulphasalazine.

### RNA isolation

Blood was collected in a PAXgene tube (PreAnalytix, Hombrechtikon, Switzerland) and frozen at −20°C until RNA isolation. Total RNA was isolated using the PAXgene blood RNA isolation kit according to the manufacturer’s protocol. RNA quantity and purity was determined using the Nanodrop spectrophotometer (Nanodrop Technologies, Wilmington, DE, USA). In case of subsequent quantitative (q)PCR measurements, RNA was converted to cDNA using the Revertaid H-minus cDNA synthesis kit (MBI Fermentas, St. Leon-Rot, Germany), according to the manufacturer’s protocol.

### Gene expression measurements and calculation of the IFN-score

The IFN-score was calculated as the mean of the log2-transformed expression values of a set of IRGs for an individual patient. A set of eight correlative IRGs (EPSTI1, HERC5, IFI44L, ISG15, LY6E, MX1, MX2 and RSAD2), previously shown to be predictive for the response to RTX [3], was measured, unless indicated otherwise. IRG expression levels were determined by DNA microarray, multiplex qPCR (Fluidigm Corporation, San Francisco, CA, USA) or conventional qPCR (ABI Prism 7900HT, Applied Biosystems, Foster City, CA, USA). To combine microarray and qPCR data, data were median-centered as described previously [3].

### Statistical analysis

Based on data normality, comparison of two groups was performed using the Student’s unpaired *t*-test or Mann–Whitney *U* test. Comparison of multiple groups was performed using one-way analysis of variance (ANOVA), Kruskal-Wallis one-way ANOVA or *χ*^2^-test, where appropriate. ROC analyses were performed using non-responder status defined as dDAS28 < 1.2 as the state variable. All analyses were performed using IBM SPSS Statistics version 20.0 (IBM Corp, Armonk, NY, USA). *P* values <0.05 were considered to be significant.

## Results

### Patient characteristics

Demographic and clinical data are shown in Table 1. Whereas cohorts I and II were not significantly different except for age (*P* = 0.024), there was a significant increase in disease activity, prednisone use and prednisone dose in cohort III (comparison of all three cohorts, *P* = 0.007, *P* <0.001 and P <0.001 respectively), probably illustrating the more severe disease state in these patients, as expected. Methotrexate (MTX) use was lower in cohort III compared to cohorts I and II. No significant differences in clinical parameters were observed in any of the cohorts between prednisone users and prednisone non-users.

### Prednisone treatment and type I IFN response gene expression

**Figure 1:**
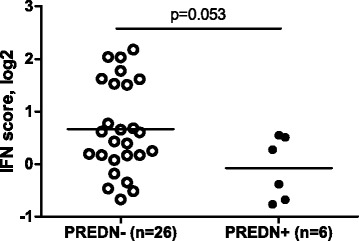
**Effect of prednisone use on IFN-score in cohort I.** In peripheral blood of 32 RA patients, gene expression levels of 7 interferon response genes were averaged to calculate the IFN-score. The IFN-score was evaluated in relation to prednisone use; prednisone-treated patients (PREDN^+^) exhibited a lower IFN-score than prednisone-untreated patients (PREDN^−^). RA, rheumatoid arthritis.

To evaluate whether prednisone use affects the type I IFN-score in RA, we initially tested the relation between prednisone use and the IFN-score in patients of cohort I. Thereto, we assessed IRG expression from available microarray data [4]. Since the *HERC5* gene was not available on the microarray at that time, the IFN-score was based on seven IRGs. This analysis revealed a difference between the IFN-score and prednisone use; the IFN-score was lower in PREDN^+^ patients compared to PREDN^−^ patients (P = 0.053, Figure 1).

**Figure 2:**
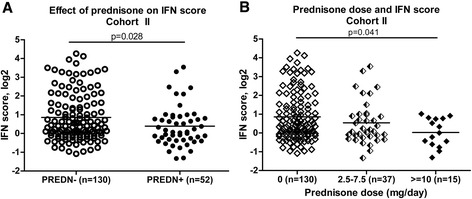
**Effect of prednisone on the IFN-score validated in cohort II.** In peripheral blood of 182 RA patients, gene expression levels of 8 interferon response genes were averaged to calculate the IFN-score. The IFN-score was evaluated in relation to prednisone use and prednisone dose. **A)** Comparison of IFN-score between prednisone-untreated (PREDN^−^) and prednisone-treated (PREDN^+^) RA patients; **B)** The relation between prednisone dose and IFN-score, assessed using Kruskal-Wallis. RA, rheumatoid arthritis.

To validate the findings from cohort I, we compared the IFN-scores between PREDN^−^ and PREDN^+^ patients in an independent cohort consisting of 182 RA patients (cohort II). This confirmed our earlier findings, showing a significantly lower IFN-score in PREDN^+^ patients compared to PREDN^−^ patients (*P* = 0.028, see Figure 2A). Overall, these findings reveal a relation between prednisone use and a low IFN-score.

In addition to variation in prednisone use itself, the dose of prednisone varied between users, from 2.5 mg/day to 20 mg/day. Therefore, we also compared prednisone dose and IFN-score. As shown in Figure 2B, the range of the IFN-score gradually decreased with increasing prednisone dose, indicating that the suppression of IFN-score is dose-dependent. The decrease in IFN-score was most pronounced at the highest doses of 10 mg/day or more (Kruskal-Wallis, *P* = 0.041).

### Effect of prednisone treatment on the predictive value of the IFN-score in the outcome of rituximab treatment

The above results indicate that prednisone use is associated with a lower IFN-score in RA. We reasoned that the suppressive effect of prednisone on the IFN-score could have implications for the clinical utility of the IFN-score as predictor for the outcome of RTX therapy in RA. Therefore, we studied the relation between prednisone use and the predictive value of the IFN-score in a cohort of 40 RA patients who were candidates for RTX therapy (Cohort III).

The predictive value towards the clinical response to RTX was determined for the eight-IRG-based IFN-score, as well as for the IFN-score based on three IRGs (EPSTI1, MX1 and RSAD2), which was previously described to give the most optimal performance as a predictor of RTX response [3]. As described before, no association of prednisone use itself as predictor for the outcome of RTX treatment was found in this cohort (odds ratio (OR):2.0, 95% confidence interval (CI):0.49 to 8.20, *P* = 0.335 [3]). As a measure of accuracy of the IFN-score in separating responders and non-responders, we performed ROC analysis on the whole group (n = 40, 18 responders, 22 non-responders), the PREDN^+^ group (n = 27, 13 responders, 14 non-responders) and PREDN^−^ group (n = 13, 5 responders, 8 non-responders).

**Figure 3:**
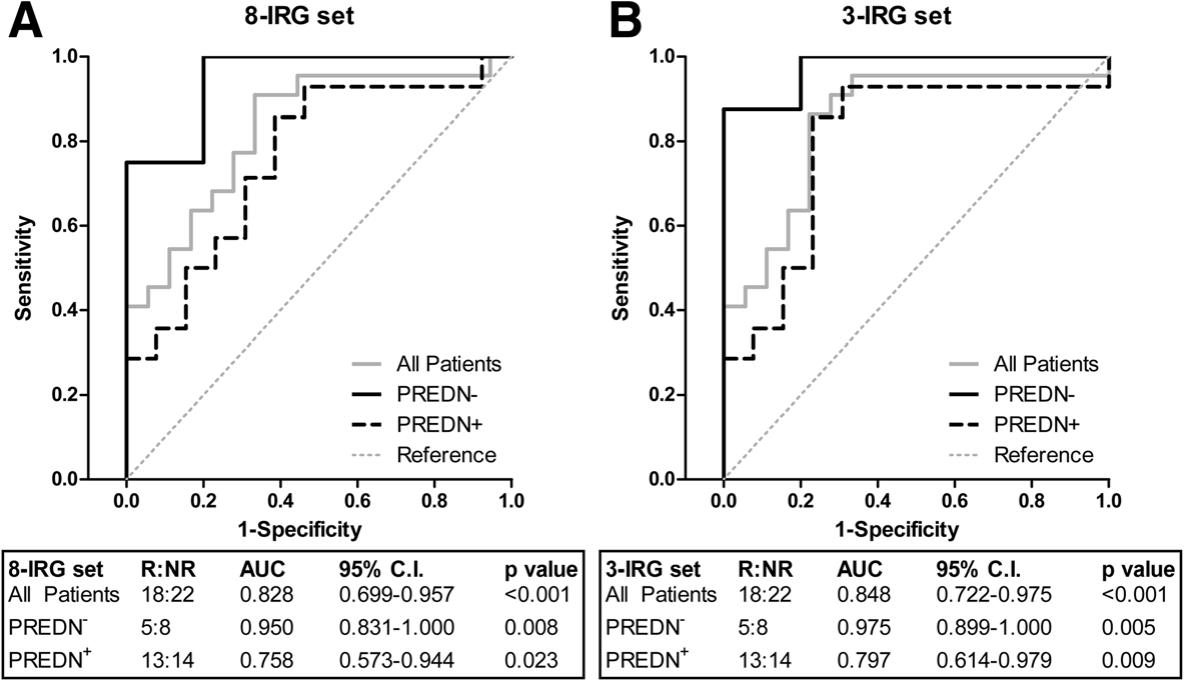
**ROC analyses of rituximab response prediction in cohort III.** The predictive value of the IFN-score for the outcome of rituximab treatment was assessed per patient subgroup based on prednisone treatment. **A)** ROC analysis for the eight-IRG set; **B)** ROC analysis for the highly predictive three-IRG set. IRG, interferon response gene; ROC, receiver operating characteristics.

For the eight-IRG set, the group as a whole showed an area under the curve (AUC) of 0.828, which is considered very good. The group of PREDN^+^ patients alone revealed an AUC of 0.758 which is less than observed for the whole group. For the PREDN^−^ patients, the AUC reached an excellent value of 0.950 (Figure 3A).

**Figure 4:**
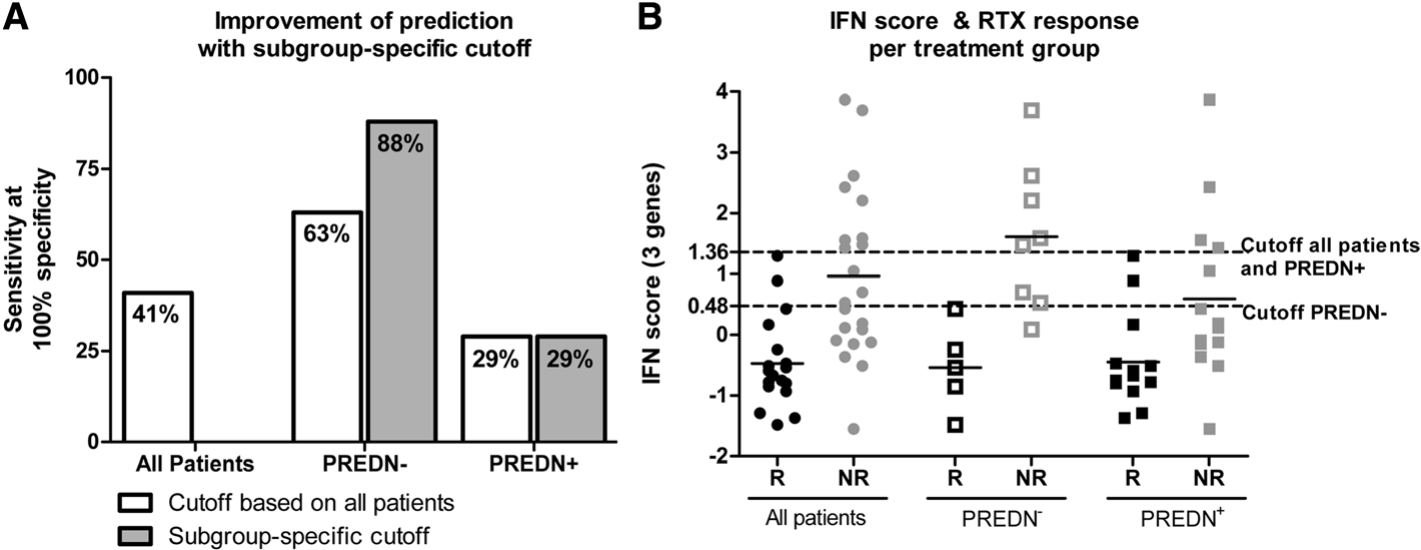
**Subgroup-specific cut-offs for rituximab prediction.** Detailed analysis of the improvement in rituximab response prediction upon stratification for prednisone use. **A)** Sensitivities of rituximab response prediction combined with 100% specificity, when using subgroup-specific cut-offs of the IFN-score, based on the ROC analyses per subgroup; **B)** IFN-scores per responder group and treatment subgroup. Subgroup-specific cut-offs for 100% specificity are indicated with the dotted lines and are 1.36 for all patients and the PREDN^+^ patients, and 0.48 for the PREDN^−^ patients. PREDN, prednisone; ROC, receiver operating characteristics.

The same ROC analyses for the optimally performing three-IRG set revealed an AUC of 0.797 in the PREDN^+^ group, again less than the AUC of 0.848 that was observed for the whole group. The PREDN^−^ group reached an AUC of 0.975, which is equivalent to an excellent prediction (Figure 3B). At an IFN-score cutoff with a specificity of 100%, this corresponds to a sensitivity of 88% in the PREDN^−^ group, compared to a sensitivity of 41% in the whole group (Figure 4A). These findings indicate that stratification on prednisone use before measuring the expression of IRGs to predict the clinical outcome of RTX treatment could dramatically improve the predictive power of the test.

A detailed analysis of the enhanced AUC in the PREDN^−^ group revealed that the improved prediction of RTX response is a consequence of a larger difference in IFN-score between responders and non-responders in the PREDN^−^ group, together with improvement of the IFN-score cut-off value. When using the IFN-score cut-off yielding 100% specificity in the whole group, the PREDN^−^ group already displayed an improved sensitivity of 63%, compared to the original 41% sensitivity of the whole group (Figure 4A). When the IFN-score cut-off was selectively determined for the PREDN^−^ group, sensitivity in this group was even further enhanced to 88% (Figure 4A and 4B). Altogether, stratification for prednisone use in this cohort resulted in the correct classification of 100% of the responders and 50% of all non-responders (7/8 non-responders in the PREDN^−^ group and 4/14 non-responders in the PREDN^+^ group), compared to 100% of responders and 41% of the non-responders (9/22 non-responders) without stratification.

## Discussion

Previous studies have shown that the IFN-score has clinical relevance by predicting the outcome of RTX therapy; a high IFN-score reflecting increased IRG expression at baseline is associated with a poor clinical response to RTX [2,3]. In the present study, we demonstrated that the average IFN-score was consistently lower in prednisone-using patients compared to patients not using prednisone. As a consequence, RTX response prediction based on the IFN-score was considerably improved when stratifying patients based on prednisone use. ROC analysis of the PREDN^−^ group based on an IFN-score of three IRGs yielded an almost perfect AUC of 0.975, compared to 0.848 and 0.797 in all patients or prednisone users alone, respectively. This means that a test based on the three-IRG IFN-score would correctly classify 98% of two PREDN^−^ patients of two randomly drawn pairs, which is considered ‘excellent.’ Based on these data, non-response to RTX could be predicted with a specificity of 100% and a sensitivity of 88% in PREDN^−^ patients.

At the moment, the IFN-score-based RTX prediction model seems to be the most discriminative test for RTX response prediction and has already demonstrated clinical utility [3,11]. The current data show that the model could be further optimized via stratification for prednisone use. GC therapy has proven to be a vital part in the management of RA [1] and is often prescribed as bridging therapy in between biologics to prevent or suppress inflammatory flares. The observation that the RTX response prediction reached optimal predictive value in patients without current prednisone treatment suggests that the prednisone-related suppression of the IFN-score obscures the ‘genuine’ intrinsic IRG expression, leading to the lower accuracy of prediction. Since elimination of prednisone use in RA patients would be practically intolerable, implementation of prednisone use and/or dose into the IFN-score-based prediction model might be an approach to optimize prediction of treatment outcome. Eventually, it might be considered to taper or temporarily stop prednisone treatment to measure the ‘genuine’ intrinsic IRG expression to predict the response to RTX treatment, if the clinical condition of the patient allows that. The current data provide the first indication of the effect of medication history on response prediction. The study consisted of cross-sectional data and results need to be confirmed in a larger prospective cohort of RA patients who are sampled before and after GC treatment. Moreover, besides medication history, the influence of cumulative dosing and term of prednisone treatment should also be analyzed.

Our findings on the *in vivo* suppressive effect of prednisone on IRG expression in RA corroborates results from mechanistic studies that reported an effect of GCs on the type I IFN system. In systemic lupus erythematosus (SLE), methylprednisolone injection coincided with a decrease in plasmacytoid dendritic cells (pDCs), which are considered to be the main producers of IFNα in SLE [12,13]. In RA, evidence is available for a role of both IFNα and IFNβ [14,15], indicating a broader cellular origin for these IFNs, making it unlikely that the prednisone-related IRG suppression in RA is caused solely by a decrease in pDCs.

Since GCs are able to interfere with the IRF3 and IRF9 pathways, thereby affecting IFNα/β induction and/or downstream IFN receptor (IFNAR) signaling, this could lead to suppression of both type I IFN production as well as downstream IRG induction. Such suppression is caused by the interaction of GRIP1/NCOA2 –a cofactor of GC signaling– with IRF3 and IRF9, and subsequent interference between GR signaling and TLR signaling and IFNAR signaling, respectively [6,7]. Additionally, it was demonstrated that GCs are able to induce expression of SOCS1 [16], a well-known inhibitor of JAK-STAT signaling, including type I IFN signaling [17]. Because both TLR and JAK-STAT signaling are implicated in the regulation of type I IFN activity in RA [18,19], this may be an additional mechanism of the observed prednisone-related IRG suppression. However, our study was not aimed at unravelling the mechanisms of GC-mediated type I IFN suppression, which is the objective of future studies.

Our observations raise questions regarding the relation between high baseline IRG expression and a poor response to RTX. It is yet unclear whether high IRG expression is (in)directly causative for RTX non-response or whether it is a related epiphenomenon. In the case of a causative relation between high baseline IRG expression and RTX non-response, it would be expected that prednisone use, as a suppressor of IRG expression, would lead to more responders. This was not observed in our cohort, as reflected by the described absence of a direct relation between prednisone use and the clinical response to RTX [3]. Moreover, we did not observe any bias in clinical parameters between the subgroups of prednisone use and RTX response. Since the numbers of patients per subgroup are rather small, this could be due to a lack of power. However, our data indicate that the difference in prediction accuracy between PREDN^−^ and PREDN^+^ patients is selectively due to prednisone-related IRG suppression in RTX non-responders, resulting in false-positive good responders in the PREDN^+^ group, whereas responders are almost perfectly distinguishable from non-responders in the PREDN^−^ group. Altogether, these observations indicate that IFN^high^ patients using prednisone might appear as IFN^low^ patients due to the prednisone-related IRG suppression, but still turn out to be non-responders to RTX. This would in turn imply that the relation between high IRG expression and RTX non-responders is not a directly causative one.

Besides the association between baseline IRG expression and RTX response, there are indications of pharmacodynamic differences during RTX therapy as well. Vosslamber *et al*. provided evidence that RTX responders, that is, patients with low baseline IRG expression, exhibited IRG upregulation after three months of therapy, whereas RTX non-responders did not [20], suggesting that type I IFN dynamics is related to the clinical outcome of RTX treatment. It was hypothesized that high IRG expression before RTX treatment could reflect an over-stimulated type I IFN system, incapable of further inducing the IRG expression that would be essential to reach a favorable response to RTX. With regard to prednisone interference, one could speculate that this process of pathway saturation, possibly caused by extensive negative feedback or shortage of signaling proteins, could be synergistically enhanced by prednisone. This would then result in the absence of IRG induction during RTX therapy, despite relatively low IRG expression at baseline. Interestingly, the majority of patients in the study of Vosslamber *et al*. was using prednisone (82%), and patients were allowed to continue using it during RTX therapy [20]. Moreover, the observed pharmacological induction of IRG expression during RTX therapy was described to be irrespective of clinical parameters, such as prednisone use [20], suggesting it was persistent despite GC interference. This could in turn imply that the IRG expression as induced during RTX treatment occurs via a different mechanism than the IRG expression at baseline, which has appeared sensitive to prednisone interference.

Our data might also be useful for other treatment regimens, as the relation between the IFN system and treatment response does not seem to be restricted to RTX. For example, for anti-TNF therapy with infliximab, the dynamics of IRG expression appeared to be related to the clinical response, as non-responders showed an IRG upregulation during treatment whereas good responders did not [21,22]. Furthermore, a genome-wide expression study revealed that high IRG expression before the start of treatment with tocilizumab, an IL-6R blocker, is associated with a favorable response [23]. The response patterns observed for these biologics are not in line with that for RTX. Although the clinical relevance for these results needs to be validated in independent studies, it may indicate that the status of the IFN system might have different clinical consequences in RA depending on the specific biologic that is used, that is, the immune pathway that is modulated. Our findings on *in vivo* interference of the IFN-system by prednisone may be equally relevant for the other biologic therapies and indications that are characterized by differential IFN activity. In these cases separate analysis of PREDN^−^ and PREDN^+^ patients could provide supportive value for these claims.

## Conclusions

In conclusion, we have demonstrated that type I IFN activity in RA patients is suppressed in prednisone users. Consequently, our findings reveal that the IFN-score based model to predict the clinical outcome of RTX treatment can be optimized by implementation of prednisone use. This result provides an accurate system for response prediction of RTX, thereby taking the paradigm of personalized medicine one step further.

## Abbreviations

ACPA: anti-cyclic citrullinated protein antibody
AUC: area under the curve
CI: confidence interval
CRP: C-reactive protein
DAS28: 28-joints disease activity score
DMARDs: disease modifying anti-rheumatic drugs
ESR: erythrocyte sedimentation rate
GC: glucocorticoid
HCQ: hydroxychloroquine
IFN: interferon
IRG: interferon response gene
MTX: methotrexate
PREDN: prednisone
RA: rheumatoid arthritis
RF: rheumatoid factor
ROC: receiver operating characteristics
RTX: rituximab
SSZ: sulphasalazine
TNF: tumor necrosis factor
